# Factor H’s Control of Complement Activation Emerges as a Significant and Promising Therapeutic Target for Alzheimer’s Disease Treatment

**DOI:** 10.3390/ijms25042272

**Published:** 2024-02-14

**Authors:** Iris Hasantari, Nabil Nicolas, Philippe Alzieu, Léa Leval, Andree Shalabi, Sylvain Grolleau, Virginie Dinet

**Affiliations:** 1 INSERM (Institut National de la Santé et de la Recherche Médicale), Biologie des Maladies Cardiovasculaires, U1034, University Bordeaux, F-33600 Pessac, France; iris.hasantari@u-bordeaux.fr (I.H.); nabil.nicolas@u-bordeaux.fr (N.N.);; 2 Medizinische Hochschule Hannover, Abt. Infektiologie, 30625 Hannover, Germany

**Keywords:** complement system, Alzheimer’s disease, factor H, therapeutic hope, cognitive functions

## Abstract

The complement is a component of the innate immune system designed to fight infections and tissue- or age-related damages. Complement activation creates an inflammatory microenvironment, which enhances cell death. Excessive complement inflammatory activity has been linked to alterations in the structure and functions of the blood–brain barrier, contributing to a poor prognosis for Alzheimer’s disease (AD). In the AD preclinical phase, individuals are often clinically asymptomatic despite evidence of AD neuropathology coupled with heightened inflammation. Considering the involvement of the complement system in the risk of developing AD, we hypothesize that inhibiting complement activation could reduce this inflammatory period observed even before clinical signs, thereby slowing down the onset/progression of AD. To validate our hypothesis, we injected complement inhibitor factor H into the brain of APP/PS1 AD mice at early or late stages of this pathology. Our results showed that the injection of factor H had effects on both the onset and progression of AD by reducing proinflammatory IL6, TNF-α, IL1β, MAC and amyloid beta levels. This reduction was associated with an increase in VGLUT1 and Psd95 synaptic transmission in the hippocampal region, leading to an improvement in cognitive functions. This study invites a reconsideration of factor H’s therapeutic potential for AD treatment.

## 1. Introduction

Alzheimer’s disease (AD) is an increasingly prevalent pathology associated with the aging of the world. The number of affected populations is often underestimated due to late diagnosis, which is currently based on scoring clinical symptoms, including the loss of cognitive functions, such as trouble remembering recent events, and eventual total memory loss, that interfere with individuals’ ability to perform daily tasks. Most neurodegenerative diseases, including AD, share common processes such as angiopathy and vascular dementia, which are associated with inflammation. These processes ultimately lead to neuronal death, resulting in cognitive impairments. The two major hallmarks of AD are the accumulation of extracellular deposits of amyloid beta (Aβ) into plaques and abnormal intracellular or hyperphosphorylated tau protein (neurofibrillary tangles) in brain regions related to memory [1]. Prior to neuronal loss, the synapse has consistently been considered a vulnerable and critical target within AD. This dysregulation of synapses, fundamental in learning and memory processes, can lead to widespread neurophysiological dysfunction. In AD post-mortem brain tissue, we note that synapse loss correlates well with cognitive abnormality [2]. Alterations and loss of several synaptic markers are observed, associated with changes in the structure and type of synapse, leading to cognitive function impairments [3]. Synaptic receptor expression is also involved in the regulation of synaptic transmission in AD, including a reduction in the expression of glutamate receptors [4].

Currently, there is no real treatment for AD. Previous treatments have focused on addressing the consequences rather than the causes of the disease. Treatment strategies encompass a range of approaches, including comprehensive medical and psychosocial support, as well as caregiving [5]. Therefore, nonpharmacologic measures and behavioral treatments are the first lines of cure. For several decades, treatment strategies targeting Aβ protein have been developed around immunization with Aβ itself. This approach has shown improvement in cognitive functions and the subsequent reduction of pathology in AD models [6]. Recent clinical trials of lecanemab (Leqembi) or donanemab in AD provide evidence that reducing brain Aβ has a clear benefit to cognitive functions, with improvements reaching up to 27% [7] and 35% [8], respectively, consistent with the amyloid hypothesis [9]. New lines of evidence from research studies and clinics worldwide support the concept that the regulation of Aβ peptide production and clearance is often involved in the onset and progression of AD. Aβ deposits are observed around neuronal and glial cells in brain regions responsible for memory and cognitive functions. Moreover, in rodent AD models, Aβ enhances long-term synaptic depression in the hippocampus [10], and the intraventricular injection of Aβ oligomers in healthy adult mouse brains impairs their cognitive functions [11], suggesting a remarkably important role of Aβ in the progression of AD. However, anti-Aβ treatments are not effective for all patients (mild cognitive impairment or at an early stage of the disease) and carry some risks (the main adverse event identified is edema in the brain) [7].

Hence, it has been reported that AD pathology settles in the brain 15 to 20 years before presenting clinical signs [12]. This early-stage formation presents cerebral neuroinflammation that, over time, becomes uncontrollable. This becomes a favorable factor for the installation of the pathology, leading to neuron loss and associated cognitive function impairments. In this preclinical phase, individuals are often clinically asymptomatic but show evidence of AD neuropathology [13]. Several studies have noted that the pathological changes characterizing AD could all result from complement activation in neuritic Aβ plaques; since the complement system activates various cell types with the release of cytokines, it alters cellular functions and induces cell damage [14,15,16]. The Aβ plaques are associated with inflammation, such as activated complement protein deposits. Both events could reflect an aggressive level of activity, which might be an important contributor to AD pathogenesis [17,18]. Among the genetic risks for developing AD are polymorphisms for complement proteins, CR1 (Complement Receptor 1) or C1s (Complement protein 1s), which are inducers of complement activation [19,20]. Interestingly, there are some indications that a Y402H polymorphism in domain 7 of factor H (FH), the main inhibitor of complement activation, could also be associated with AD, although conflicting results on this association have been found in different genetic studies [21,22,23]. In addition, it has been shown that overactivation of this pathway is an aggravating factor in AD [17,18].

The complement system is a collection of blood and cell surface proteins grouped together to be activated. Activated complement acts as a powerful amplifier of both innate and acquired immunity by increasing antibody responses and immunologic memory. It functions by lysing cells, clearing immune complexes and apoptotic cells. The major event leading to the activation of the three complement pathways (classical/lectin or alternative) is the cleavage of C3 (Complement protein 3) by a C3 convertase complex into a C3a anaphylactic protein and a C3b opsin protein (Supplemental Figure S1a). The latter is involved in phagocytosis and the formation of the C3/C5 convertase complex. The C5 convertase complex induces the cleavage of C5 (Complement protein 5) into an anaphylactic/chemotactic C5a product and a C5b molecule, which participates in the formation of a membranolytic complex (C5b-9 or MAC (complement Membrane Attack Complex), Supplemental Figure S1a). In tissue lesions, C3a/C5a, C3b and MAC serve as markers of activated complement, functioning as mediators in local inflammatory processes. Activation of the complement system must be tightly regulated, as it has the potential to be extremely damaging to host tissues. The main soluble inhibitor of the complement alternative pathway is complement factor H (FH), a 155kda sushi protein that acts in body fluids and on cell surfaces. FH prevents the formation and accelerates the decay of the C3/C5 convertases and assists in the degradation of C3b, thereby breaking the complement-positive C3 convertase complex feedback loop (Supplemental Figure S1a). FH may also regulate complement-independent processes such as apoptosis, angiogenesis or oxidative stress, which are involved in the development of AD [24,25]. In the brain of AD patients, the levels of C3, the major compound of complement activation, are upregulated [26]. In AD mice models, blocking this protein rescues synapse loss, improving neurophysiological and behavioral measurements [26,27]. Previous studies of the C5aR signaling pathway indicate that the anaphylactic C5a produces a proinflammatory microenvironment, leading to the generation of cytokines, many of which are known to be implicated in AD progression [28,29,30]. Altogether, these data clearly show a role of complement activation in the progression of AD.

Considering the role played by complement in the pathogenesis of AD, we hypothesize that complement overactivation may participate in the onset and/or progression of AD disorders and that the inhibition of complement activation by FH could prevent or treat AD.

Our data point to improvements in cognitive functions after FH injection in the APP/PS1 cerebrospinal fluid (CSF) during both early and late stages. These results concur with the reduction in inflammation and Aβ deposits, as well as with the enhancement of synaptic transmission.

## 2. Results

### 2.1. Factor H Plays a Protective Role against Complement-Induced Lysis in Blood Endothelial Cells

**Figure 1:**
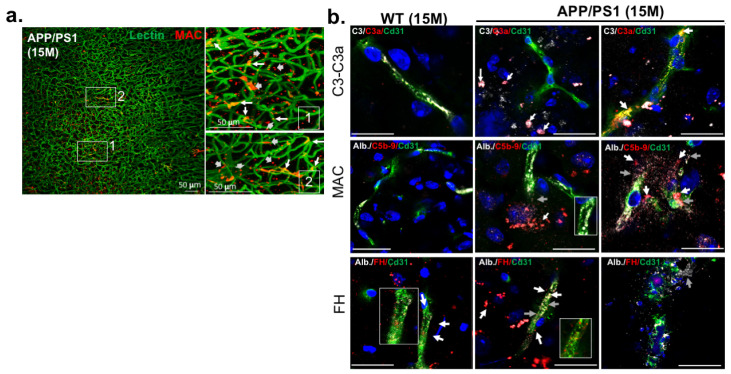
FH localization on brain APP/PS1 vessels at a late stage of AD (15 months). (**a**) Optical clearing experiments: MAC (C5b-9, red) was detected in/on/around brain vessels of APP/PS1 mice immunostained by the lectin marker (green). (**b**) Immunostaining experiments: C3 (white), C3a (red) and MAC (C5b-9, red) were markers of complement overactivation observed here in brain blood vessels (Cd31, green) of APP/PS1 mice. Complement overactivation resulted in albumin leakage (white). In contrast, deposits of FH (red) on brain vessels (Cd31, green) may provide protection against albumin leakage. *n* = 5 to 7 animals per experimental group. Scale bar = 50 µm, Alb. = Albumin.

Complement cascade activation has been previously identified in many pathological states, including vascular disorders associated with neurodegeneration, two processes implicated in AD progression. Since one of the major events in AD pathogenesis is small vessel disease (SVD), we investigated complement activation in APP/PS1 network brain vessels at the late stage of pathology (15 Months). Our data show significant deposits of the activated complement marker, MAC (C5b-9), in/around and on the brain vessels (Figure 1a), suggesting that vessel alterations could be induced by an overactivation of the complement system. This was also confirmed by significantly higher levels of C3 and C3 cleaved fragments (C3a) on brain endothelial cells in APP/PS1 mice compared to WT mice (Figure 1b). In addition, we demonstrated that lysed brain vessels with high levels of MAC staining coincided with albumin leakage (Figure 1b), suggesting that complement overactivation could contribute to AD-breached vascular wall through MAC lysis function. Interestingly, no albumin vessel leakage was observed in endothelial cells presenting strong FH deposits (Figure 1b). Altogether, these data suggest that the accumulation of FH on endothelial cells could prevent complement overactivation, thus preventing the blood vessel damage observed in AD.

**Figure 2:**
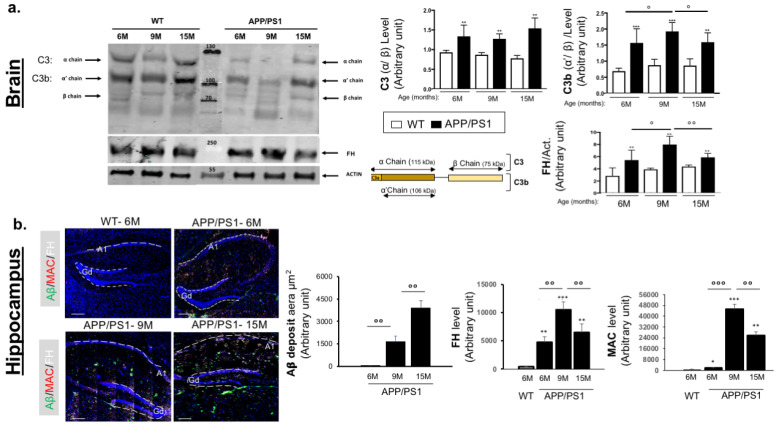
Complement activation markers and FH kinetic immunostaining in the brain and hippocampus of APP/PS1 vs. WT mice. (**a**) Western blotting experiments were used to characterize the C3 cleavage and FH expression in the total brain of APP/PS1 vs. WT mice between 6 and 15 months (*n* = 10 mice). The ratio of C3- α′cleaved chain/C3- β chain represents the production of C3b while the ratio C3- α chain/C3- β chain represents uncleaved C3. (**b**) Immunostaining experiments analyzed MAC (red), FH (white) and Aβ (green) production in the hippocampus of APP/PS1 mice vs. WT at 6, 9 and 15 months (*n* = 5 to 7 mice). For both the hippocampus immunostaining and brain Western blot experiments, semiquantitative analyses were performed. As WT mice do not produce human Aβ and the antibody used (6E10) recognizes human Aβ, we considered the Aβ signal observed in WT mice tissue as zero, and then we subtracted this signal level from the APP/PS1 signal level. Results were expressed as the staining intensity ± the standard error of the mean (SEM). Spatial frequency threshold across ages and genotypes (C57BL/6J (WT) vs. APP/PS1) was analyzed using the Mann–Whitney U test. *p*-values of 0.05 or less were considered significant. (°^,^* *p* < 0.05; °°^,^** *p* < 0.01; °°°^,^*** *p* < 0.005). Scale bar = 50 µm.

Dysregulation, impairment or inadvertent activation of complement fragments as well as their regulators contribute to the inflammatory toxicity in the microenvironment of the brain. To determine the critical and optimal stage of complement overactivation in the APP/PS1 brain, we first evaluated the level of complement C3, its activated fragment (C3b) and its inhibitor FH using total brain protein extractions at 6 to 15 months, ages at which the vascular damages progress in this AD murine model [31,32]. We investigated complement activation by measuring the C3b concentration using an anti-C3 antibody that recognizes the alpha (α~115 kDa), alpha-cleaved chain (α′~110 kDa) and beta chain of C3 (β~75 kDa) (Figure 2a). The ratio of C3- α′cleaved chain/C3- β chain represents the production of C3b, while the ratio C3- α chain/C3- β chain represents uncleaved C3. Compared to WT mice, we found a constant higher concentration of uncleaved C3 in the brain of APP/PS1 at all stages studied (Figure 2a), suggesting an upregulation of C3 in the brain of APP/PS1. Concerning C3b, we also observed a higher production in the brain of APP/PS1 mice vs. WT, with a maximum observed at 9 months (Figure 2a), demonstrating an optimal complement activation system at 9 months in the brain of APP/PS1 mice. Increased levels of C3b were accompanied by increased production of FH in the brain of APP/PS1 between 6 and 9 months, but it significantly decreased between 9 and 15 months (Figure 2a), suggesting a slight inhibition of the complement system in the later stages. We then focused on hippocampal tissue, the major target of AD, where we observed a high level of activated complement MAC starting at 6 months, reaching its optimum at 9 months (Figure 2b). These data were associated with a decrease in FH concentration between 9 and 15 months in APP/PS1 mice, consistent with an overactivation of complement (Figure 2b). In parallel with overactivation of complement, our data show a significant increase in Aβ deposits starting at 9 months in the hippocampus of APP/PS1 mice (Figure 2b). Together, all these data provide evidence that the overactivation of complement in the hippocampus of APP/PS1 mice starts at 6 months (early stage) before the deposits of Aβ and reaches its optimum at 9 months (late stage), despite the increase in FH levels between 6 to 9 months. Based on these results, we hypothesized that injecting a high concentration of FH could decrease complement activation at an early stage (early: 6 months) or later stage (late: 9 months) and thus could protect the hippocampus from irreversible complement overactivation damage leading to AD cognitive impairments.

With the aim to evaluate the human potential therapeutic effect of FH in AD and since the APP/PS1-AD mouse model expresses two mutated human transgenes, we have chosen to inject human plasmatic FH (plFH). This form of FH contains all expected human glycosylations. Moreover, we have previously shown that the injection of human plFH is active in regulating endogenous complement activation in mice by reducing the cleavage of C3 into C3b [25]. A quantity of 50 µg of plFH was chosen based on our previous results obtained for the treatment of laser-induced age-related macular degeneration in mice [25]. We decided to inject plasmatic human FH into the cerebrospinal fluid (CSF) of APP/PS1 mice at two stages of AD development: the early stage (6 months) and the late stage (9 months) (Supplemental Figure S1b,c). The injections in the CSF were favored over systemic injections since the latter may lead to lower host immune defense and avoid the blood–brain barrier function, which is impermeable to large molecules such as FH (155 kDa). The ependymal cells, which constitute the ventricles and ducts carrying the CSF, present tight junctions only on the choroidal plexuses interface and looser junctions for the other regions of the brain, which increase its exchanges with cerebral tissues. So, at first, we confirmed with a CSF Evans blue injection that the plFH stereotaxic coordinates targeted the left lateral ventricle. Secondly, we determined the fate of exogenous injected human plFH by a single injection in the CSF of a histidine-tagged FH (FH-HisTag). We confirmed that FH-HisTag diffused into the hippocampus and cortex regions of APP/PS1 at 24 h and one month post-injection (Supplemental Figure S2a). In contrast, no more FH-HisTag was detected 3 months post-injection (Supplemental Figure S2a). Finally, we showed that the injection of plFH in the brain of WT mice did not lead to cognitive dysfunctions (Supplemental Figure S2b). Given our results, we decided to evaluate the therapeutic effect(s) of plFH in early-stage (6 months) and late-stage (9 months) APP/PS1 mice at 1, 3 or 6 months post-injection.

### 2.2. Factor H Transiently Improves Cognitive Functions in Early-Stage AD-APP/PS1 Mice

**Figure 3:**
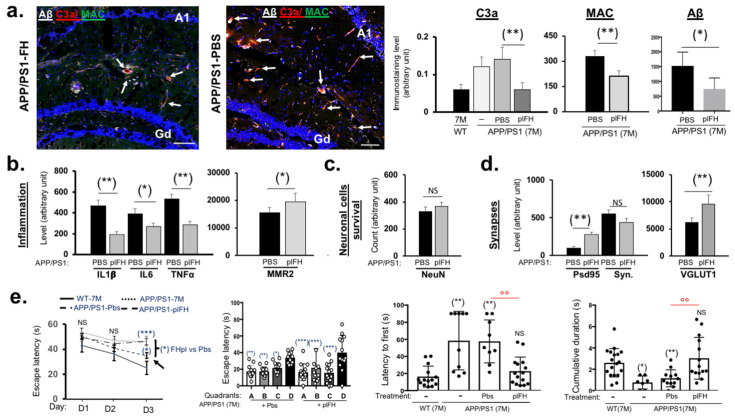
Analysis of plFH injection effects on the hippocampus of APP/PS1 (6 months) one month post-injection (early time). Immunostaining experiments: (**a**) plFH not only reduced complement activation (MAC formation and C3a cleavage product) but also Aβ deposits in the hippocampus of APP/PS1 vs. PBS-injected APP/PS1 mice. Analysis of the impact of plFH injection on (**b**) inflammatory responses, (**c**) neuronal cell survival and (**d**) synaptic transmission in the hippocampus of APP/PS1 vs. PBS-injected APP/PS1 mice. The white arrows represent the colabelling of MAC and C3a. *n* = 3 to 7 mice per injected group (plFH or PBS). Results were expressed as the immunostaining intensity ± the standard error of the mean (SEM). Spatial frequency threshold across ages and genotypes (C57BL/6J (WT) vs. APP/PS1 vs. APP/PS1 injected with plFH or PBS) was analyzed using the Mann–Whitney U test. *p*-values of 0.05 or less were considered significant. * *p* < 0.05; ** *p* < 0.01; *** *p* < 0.005 and NS = nonsignificant. Scale bar = 50 µm. Gd = Gyrus dentatus (dentate gyrus) of the hippocampus. Morris water maze experiments: (**e**) injection of plFH in the APP/PS1 brain significantly improved escape learning time, quadrant and platform searching as well as cumulative duration searching compared to the PBS-injected groups. *n* = 10 to 15 animals for each experimental group (APP/PS1 not injected mice, plFH-injected APP/PS1 mice, PBS-injected APP/PS1 mice and WT mice). Each data point corresponds to a measurement taken from one mouse. Data are subjected to an analysis of variance (2-way ANOVA) followed by the post hoc test. Values are expressed as mean ± SEM. *p*-value < 0.05 was acknowledged for the minimal significance level. * *p* < 0.05; °°^,^** *p* < 0.01; *** *p* < 0.005 and NS = nonsignificant. “-” = noninjected mice.

plFH effects on APP/PS1 mice were determined by investigating levels of Aβ deposits, proinflammatory markers, survival cell death markers, synaptic transmission and cognitive functions. Our results showed that one month post-injection in 6-month-old APP/PS1 mice, plFH significantly reduced complement activation, as indicated by decreased levels of both C3a and MAC in the hippocampus (Figure 3a), demonstrating the efficiency of human plFH on endogenous complement regulation in mice. Moreover, we observed a significant decrease in Aβ deposits in plFH-injected mice at 7 months (6 months + 1 month post-injection) compared to APP/PS1 mice injected with PBS (Figure 3a). Additionally, plFH injection modulated the inflammatory microenvironment, evidenced by a decrease in TNF-α, IL6 and IL1β levels in the hippocampus compared to APP/PS1 mice injected with PBS (Figure 3b and Supplemental Figure S3). Furthermore, our results showed an increase in macrophage (type 2: MMR2) infiltration in APP/PS1 plFH-treated mice compared to PBS-treated animals (Figure 3b and Supplemental Figure S3). The NeuN immunostaining showed no difference between plFH- and PBS-injected APP/PS1 mice (Figure 3c). Concerning synaptic transmission, we observed a significant increase in VGLUT 1 and Psd95 levels in plFH-injected APP/PS1 mice compared to PBS-treated mice (Figure 3d and Supplemental Figure S3). Taken together, the effects of plFH on inflammation and synaptic transmission led to the improvement of cognitive functions. Indeed, we observed better learning in APP/PS1 mice injected with plFH, with a notable ability to find the platform more quickly after 3 days of learning than mice injected with PBS (Figure 3e). In addition, plFH-injected APP/PS1 mice promptly found the area of the platform and spent more time searching for this ghost platform than PBS-injected mice (Figure 3e, Table 1), suggesting that FH injection significantly improves spatial memory in APP/PS1 during the early stage. Three months after plFH injection in APP/PS1 mice, despite a decrease in Aβ and MAC levels, we did not observe further improvement in synaptic transmission and cognitive functions (Supplemental Figure S4). This correlated with no modulation of the inflammatory microenvironment compared to PBS-injected mice (Supplemental Figure S4, Table 1). Altogether, our results clearly demonstrate a transient therapeutic effect of FH in APP/PS1-AD mice during the early stages.

### 2.3. Factor H Improves the Cognitive Functions of 9-Month-Old APP/PS1 Mice up to 6 Months Post-Injection

**Figure 4:**
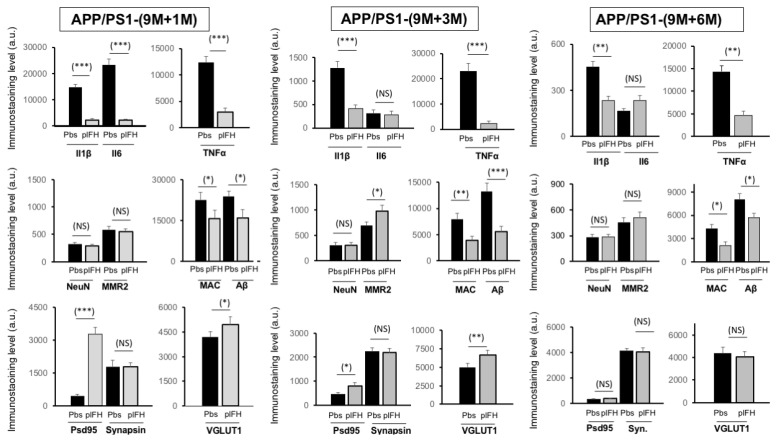
Immunostaining semiquantitative analysis of plFH injection effects on the hippocampus of APP/PS1 at 1, 3 and 6 months post-injection (late time). The analysis evaluated the impact of plFH injection on the expression of inflammation markers (IL1β, IL6, TNF-α and MMR2), MAC (C5b-9) and Aβ deposits, neuronal cell survival (NeuN) and synaptic transmission (Psd95, synapsin and VGLUT1) in the hippocampus of APP/PS1 vs. PBS-injected APP/PS1 mice (*n* = 5 to 7 mice for each immunostaining experiment). Results were expressed as the immunostaining intensity ± the standard error of the mean (SEM). Spatial frequency threshold across ages and treatments (APP/PS1 injected with plFH or PBS) was analyzed using the Mann–Whitney U test. *p*-values of 0.05 or less were considered significant. * *p* < 0.05; ** *p* < 0.01; *** *p* < 0.005 and NS = nonsignificant.

Since our results demonstrated a transient therapeutic effect of FH in early APP/PS1 mice post-injection, we then wanted to confirm these results in late-stage APP/PS1 mice (9 months). Our results show that human plFH injection leads to the inhibition of complement activity by a decrease in C3a levels in APP/PS1 (9 months) mice vs. PBS-injected animals 1 month post-injection. As previously observed, a decrease in Aβ and MAC deposits was also measured in plFH- vs. PBS-injected APP/PS1 mice at all post-injection stages (Figure 4 and Supplemental Figure S5), confirming the regulatory role of FH on Aβ accumulation and complement activation. One, three or six months after plFH injection in 9-month-old APP/PS1 mice, our results show a significant decrease in TNF-α and IL1β levels in the hippocampal region compared to PBS-injected experimental groups (Figure 4 and Supplemental Figure S5). We noted that immunostaining of IL6 decreased only one month after plFH injection in APP/PS1 mice compared with other post-injection times (Figure 4 and Supplemental Figure S5), suggesting a specific inflammatory microenvironment at this time point. In parallel, only at 3 months post-injection time did we note a significant increase in MMR2 macrophage infiltration in plFH-injected APP/PS1 mice compared to the PBS-injected groups (Figure 4 and Supplemental Figure S5). For all post-injection times tested, no effect on neuronal cell survival (NeuN immunostaining) was observed in plFH-injected APP/PS1 mice compared to PBS-injected animals (Figure 4 and Supplemental Figure S5). Furthermore, at 1 month and 3 months (although not at 6 months post-injection), we observed higher levels of Psd95 and VGLUT1 in the hippocampus region of plFH-injected mice compared to the PBS ones (Figure 4 and Supplemental Figure S5).

**Figure 5:**
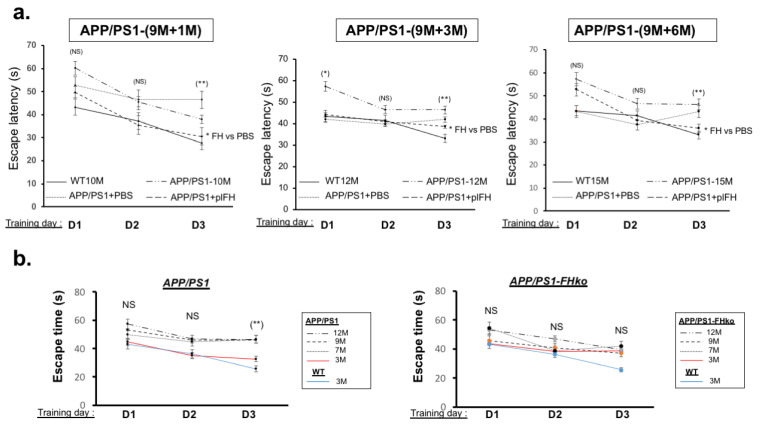
Escape learning time analysis by Morris water maze experiments: (**a**) plFH injection in APP/PS1 mice compared to APP/PS1-PBS injected/APP/PS1 and WT mice. Injection of plFH in the APP/PS1 brain significantly improved escape learning time compared to the PBS-injected mice. (**b**) APP/PS1-FHko mice compared to APP/PS1 and WT Mice. The invalidation of *fh* expression accelerated AD pathology, leading to a loss of function in escape learning time observed at 3 months compared to the APP/PS1 mice at the same age. *n* = 10 to 15 animals for each experimental group (APP/PS1 mice, plFH-injected APP/PS1 mice, PBS-injected APP/PS1 mice, WT mice and APP/PS1-FHko mice). Each data point corresponds to a measurement taken from one mouse. Data are subjected to an analysis of variance (2-way ANOVA) followed by the post hoc test. Values are expressed as mean ± SEM. *p*-value < 0.05 was acknowledged for the minimal significance level, * *p* < 0.05; ** *p* < 0.01 and NS = nonsignificant.

**Figure 6:**
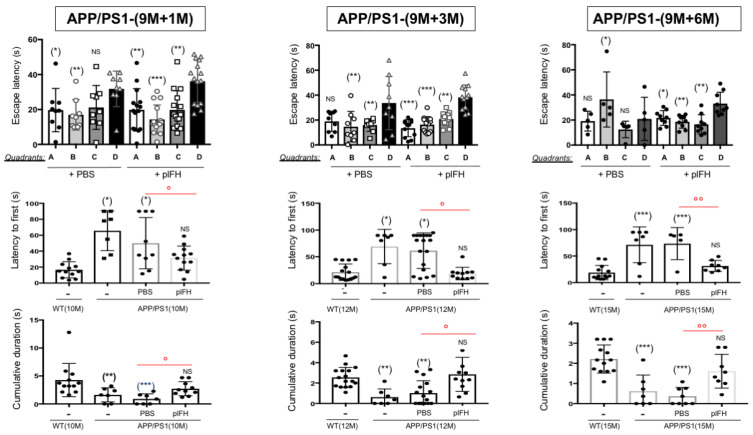
Analysis of Morris water maze experiments illustrating the effects of plFH injection on the cognitive functions of APP/PS1 at 1, 3 and 6 months post-injection (late time). Injection of plFH significantly enhanced the quadrant and platform searching, as well as the cumulative duration searching compared to PBS-injected APP/PS1 mice at all investigated time points. The study involved 10 to 15 animals in each experimental group (APP/PS1, plFH-injected APP/PS1 mice, PBS-injected APP/PS1 mice and WT mice). Each data point corresponds to a measurement taken from one mouse. Data underwent an analysis of variance (2-way ANOVA) followed by the post hoc test. Values were expressed as mean ± SEM. A *p*-value < 0.05 was acknowledged for the minimal significance level. °^,^* *p* < 0.05; °°^,^** *p* < 0.01; *** *p* < 0.005 and NS = nonsignificant. “-” noninjected mice.

In accordance with a reduction in the inflammatory response associated with synaptic transmission improvements, plFH significantly enhanced escape learning time, quadrant and platform searching, as well as the cumulative duration of searching as compared to PBS-injected or not injected APP/PS1 groups (Figure 5a and Figure 6, Table 1). All these data confirm the therapeutic effect of FH on AD including late treatment.

### 2.4. Deletion of Factor H Expression Accelerates Loss of Cognitive Functions in APP/PS1 Mice

**Figure 7:**
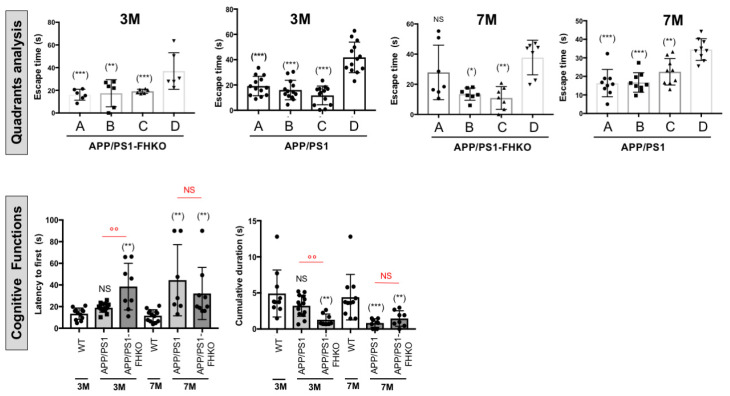
Cognitive function analysis of APP/PS1-FHko vs. APP/PS1 and WT mice at 3 and 7 months (Morris water maze experiments). The invalidation of *fh* expression accelerated AD pathology, leading to an earlier onset of cognitive function loss observed at 3 months compared to the APP/PS1 mice at the same age. The study involved 10 to 15 animals for each experimental group (APP/PS1 mice, APP/PS1-FHko mice and WT mice). Each data point corresponds to a measurement taken from one mouse. Values were expressed as mean ± SEM. Data were subjected to an analysis of variance (2-way ANOVA) followed by the post hoc test. *p*-value < 0.05 was acknowledged for the minimal significance level. * *p* < 0.05; °°^,^** *p* < 0.01; *** *p* < 0.005 and NS = nonsignificant.

Since we showed that plFH injection improved cognitive functions in APP/PS1 mice at both early (6 months) and late injection stages (9 months), we investigated the effects of FH expression invalidation on the memory of APP/PS1-FHko mice. Our results show that starting at 3 months of age, APP/PS1 mice lacking FH expression had more difficulty in learning to find the platform than APP/PS1 mice expressing FH (Figure 5b and Figure 7). However, the APP/PS1-FHko mice presented the same platform quadrant escape latency time as APP/PS1 mice (Figure 7). On the other hand, they took a longer time to escape to reach the ghost platform and found it less often within the allotted time compared to APP/PS1 mice (Figure 7), demonstrating that the loss of FH expression accelerated AD pathology. As with the APP/PS1 mice, at 7 months (corresponding to 6 months + 1 month post-injection), the APP/PS1-FHko mice ended up losing their memory capacity to quickly find the platform compared to WT mice at the same age (Figure 7). However, they faced more difficulty finding the quadrant escape compared to APP/PS1 mice (Figure 7).

At 9 and 12 months old, both APP/PS1-FHko and APP/PS1 mice lost cognitive functions in the same way (Supplemental Figure S6). Invalidation of FH expression in WT mice showed no effect on cognitive functions (Supplemental Figure S7), emphasizing that the absence of FH expression had only an aggravating effect in AD pathological environments.

## 3. Discussion

**Figure 8:**
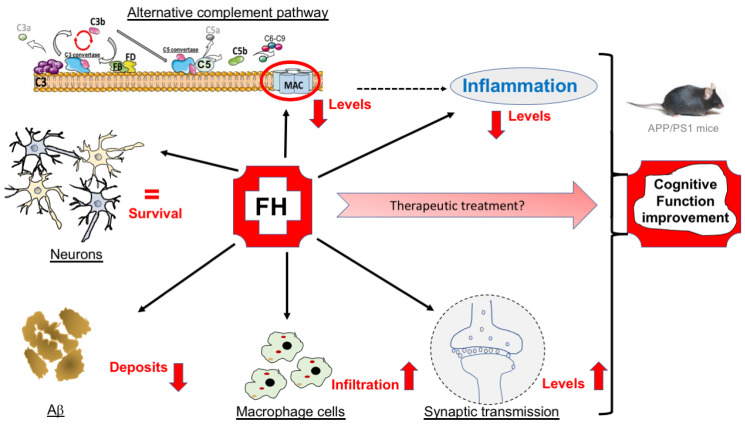
Schematic representation of the effects of an injection of FH into the brain of APP/PS1 mouse models of Alzheimer’s disease: a therapeutic hope?

This current study is the first to evaluate the effects of a single FH injection on AD’s processes including inflammation, Aβ deposits, neuronal cell survival, synaptic transmission and cognitive functions. We demonstrated that complement overactivation appears weakly at 6 months and reaches its peak at 9 months in the hippocampus of APP/PS1 mice. The concentration of FH decreases at 9 months corresponding to both the peak of complement overactivation and the emergence of high Aβ deposits in the APP/PS1 brain [32]. Furthermore, our results indicate that on brain vessels, the accumulation of MAC deposits, in contrast to FH, leads to albumin leakage, suggesting that FH plays a protective role in small vessel disease by reducing complement overactivation. In this context, our results clearly show that a single FH injection at early (6 months) or late (9 months) AD stages improves cognitive functions in APP/PS1 mice by increasing synaptic transmission and decreasing Aβ deposits, complement activation and cytokine release, demonstrating that FH could be a promising target for Alzheimer’s disease treatment (Figure 8).

Toxic Aβ aggregates are commonly found in cortical blood vessels such as cerebral amyloid angiopathy [33,34]. Typically, these deposits are also associated with inflammation, which is an important element in AD pathogenesis and significant in neurodegenerative disorders. Chronic inflammation is proposed as a dysregulated mechanism in AD patients [35]. Concomitant with these data, we observed MAC deposits on/in and around APP/PS1 brain vessels associated with vessel leakage, suggesting that complement system overactivation plays a role in AD brain vessel damage. Inflammatory mediators like IL1β, IL6 and TNF-α have also been implicated in AD progression. Mechanisms underlying early neuronal dysfunctions and transient synaptic hyperexcitability involve the increase in TNF-α during the early stage of AD [36,37,38]. Elevated levels of TNF-α in the hippocampus are associated with neuroinflammation and memory impairment in AD mice models [36,39]. Several lines of evidence using genetic and pharmacological manipulations indicate that cytokine signaling pathways, like TNF-α, exacerbate Aβ pathologies in vivo [40] and induce C3 cleavage, leading to complement overactivation [41]. In line with the potential therapeutic target of FH for treating AD, we observe a decrease in TNF-α levels associated with fewer Aβ deposits, suggesting a control by FH on TNF-α effects. IL1β also appears to play a major role in AD pathogenesis. IL1β has been reported to increase amyloid precursor protein expression [42] and exacerbate tau protein phosphorylation [43], two important markers of AD progression. Blocking IL1β signaling rescues cognition in an Alzheimer’s disease mouse model (3xTg-AD mice) [44]. Consistent with the beneficial role of FH in AD pathogenesis, we show that FH injection is able to decrease IL1β secretion in APP/PS1 mice. IL6 is a component of early-stage amyloid plaque formation in AD [45] and has been implicated in synapse loss of hippocampal neurons [46] and learning deficits in mice [47]. Neutralization of IL6 in the brain of an AD mouse model rescues memory deficits [48]. Previous meta-analyses have shown that IL6 is increased in CSF and plasma of AD patients compared to control individuals [49]. Altogether, these data suggest a real link between IL6 and AD pathogenesis. In this study, we demonstrate an early significant decrease in IL6 levels after FH injection in the hippocampus of APP/PS1 mice, suggesting a beneficial role of FH in reducing IL6 signaling and mitigating the effects of AD. Our findings establish a regulated complement system with FH as a key mechanism linking the regulation of inflammation and AD pathogenesis. Complement inhibition by FH decreases the inflammatory response associated with AD, whether FH is injected in the early or late stage of this pathology. However, a single injection of FH during the inflammatory period preceding the symptoms of AD no longer exerts any beneficial effect 3 months after injection, suggesting that the positive effects of complement inhibition in AD are transient and would require another injection.

AD is characterized morphologically by a loss of synapses and synaptic markers in the cortical and hippocampal regions. Synapse loss occurs early in the AD brain and strongly correlates with cognitive dysfunctions [50]. It has been suggested that the alteration of synaptic transmission is responsible for AD progression involving early degeneration of glutamatergic neurotransmission. Glutamate, the major excitatory neurotransmitter of the brain, is crucial for learning and memory. Several studies have reported a reduction in glutamate levels in AD patients‘ brains [51]. Among the different forms of vesicular glutamate transporters (VGLUT), VGLUT1 is expressed in the hippocampus regions and is used as a marker for glutamatergic terminals [52]. The loss of VGLUT1 and VGLUT2 in the frontal cortex correlates with cognitive dysfunctions in AD [53]. Aβ downregulates VGLUT1 in AD, impacting cognitive functions [54]. Rodriguez-Perdigon and collaborators suggest that AD progression is exacerbated by abnormal regulation of VGLUT1 [55]. Our results indicate that FH injection, in both early and late AD stages, increases VGLUT1 levels, suggesting enhanced glutamate transmission and a potential beneficial effect on AD. Postsynaptic density protein 95 (Psd95) is the most important and abundant scaffolding protein of the postsynaptic membrane and regulates synaptic transmission and plasticity. Reduced Psd95 levels are observed in the hippocampus of subjects with amnestic mild cognitive impairment [56], acting as an aggravating factor leading to cognitive decline. In agreement with the beneficial effect of FH on synaptic transmission in AD, we detect a higher level of Psd95 after FH injection. For the first time, we demonstrate that the regulation of complement overactivation by FH injection promotes synaptic transmission, which is known to be a marker of AD progression. This validates FH as a potential therapeutic target for treating AD.

AD is a multifactorial disease. Complement dysfunction may contribute to neurodegeneration decades before clinical symptoms manifest in AD. Depending on the level of activation, complement can be neurotoxic, influencing proinflammatory cytokine secretion like IL6, IL1β or TNF-α. The release of these cytokines can lead to synapse dysfunction and neuronal cell death, ultimately resulting in cognitive decline. In aging mice, mice with a *C3* gene deficiency exhibit better learning and memory in the hippocampus compared to their respective aged WT mice [57]. Additionally, APP/PS1- C3^−/−^ mice show an abundance of Aβ in the late stage of AD and perform better in cognitive tasks compared to APP/PS1-C3^+/+^ mice [27]. Despite the abundance of Aβ, there is a marked reduction in the proinflammatory cytokine TNF-α observed in APP/PS1-C3^−/−^ mice [27]. Altogether, these data support our results, showing that the inhibition of complement activation by FH injection or *C3* gene deficiency undoubtedly has a beneficial effect on AD pathogenesis. The stage at which complement activation is inhibited in AD appears crucial for achieving long-term effects. A single injection of FH during the early stage in the APP/PS1 AD mouse model improves cognitive functions only for a transient period; no effect is detected 3 months post-injection. At the late stage, cerebral administration of FH improves cognitive functions in APP/PS1 mice up to 6 months post-injection. Overall, our data suggest that complement activation inhibition should not occur too early, otherwise, its effects are limited and another injection may be needed.

## 4. Materials and Methods

### 4.1. Ethics for Animal Use

All experiments were conducted in accordance with the European Communities Council Directive 86/609/EEC and French national regulations. Adult female mice, including APP/PS1 or APP/PS1-FHko models, along with age-matched wild-type (WT) C57BL/6J control mice (Jackson Laboratory) at 3, 6, 7, 9, 10, 12 and 15 months of age were used. Also, FHko (FH^−/−^) mice provided by Dr. Pickering were well characterized [58] and included in this study. The APP/PS1 transgenic mice overexpress hAPP with mutations linked to familial AD (Swedish and Indiana mutations) coupled with a mutant human presenilin (PS1-E9) yielding vast amounts of Aβ plaques. Both mutations are associated with early-onset AD. These transgenic mice exhibit an amyloid phenotype and cognitive dysfunctions at the late stage, developing Aβ deposits around 9 months of age with an age-dependent increase in Aβ levels and amyloid plaques in the brain [32]. The accumulation of amyloid differentially affects males and females of the APP/PS1 transgenic line, with levels found to be ten-fold higher in 15-month-old females [31]. In humans, data indicate that the remaining lifetime risk of AD for a 65-year-old man is 6.3% and the remaining lifetime risk of developing any dementing illness is 10.9%. The corresponding risks for a 65-year-old woman are 12% and 19%, respectively, almost twice that of a man, highlighting the greater risk for females to develop AD dementia [59].

FHko mice were crossed with APP/PS1 mice to generate APP/PS1 (heterozygotes)/*FH*ko (heterozygotes) mice, which were backcrossed with *FH*ko mice to generate APP/PS1 (heterozygotes)/*FH*ko (homozygotes). Subsequently, these mice were bred with *FH*ko mice to generate both APP/PS1-FHko mice and littermate *FH*ko mice. A separate cohort of APP/PS1 mice was bred with C57BL/6J mice to generate WT littermates and heterozygotes APP/PS1 mice. Mice were genotyped using PCR technique with the following primers: APP/PS1: 5′-GACTGACCACTCGACCAGCTT-3′ and 5′-CTTGTAAGTTGGATTCTCATAT-3′; FH: 5′-GTAAAGGTCCTCCTCCAAGAG-3′ and 5′-GGTATAAACAACCTTTGCACC-3′ (*FH* WT); 5′-GTAAAGGTCCTCCTCCAAGAG-3′ and 5′-GGGGATCGGCAATAAAAAGAC-3′ (*FH* mutant). PCR conditions were set at denaturation at 95 °C for 1 min, annealing at 55 °C for 30 s and extension at 72 °C for 1 min for 30 cycles. All mice used in this study were housed in the laboratory animal facility (agreement number: B33-318-701, ref. 202003021406937/APAFIS N°24555). Animals were maintained under pathogen-free conditions with ad libitum access to food and water. They were housed in a 12 h/12 h light/dark cycle. For all procedures involving tissue collection, mice were first anesthetized with isoflurane (3% and 1.5% maintenance) (Aerrane^®^, Baxter, Bordeaux, France) and then euthanized by cervical dislocation.

### 4.2. Factor H Injection in Cerebrospinal Fluid Mice

Each mouse was anesthetized using a mixture of air and isoflurane (3% induction and 1.5% maintenance). Subsequently, the skull was shaved and the animal was positioned on a stereotaxis frame (Stoelting, IL, USA) and the skin was incised over 1 cm. A first subcutaneous injection of buprenorphine (0.03 mg/kg) was administered 30 min before the surgery, followed by a second injection 8 h later. Stereotactic surgery involved drilling a hole into the skull at the prefrontal medial cortex using an adapted drill. The injector was composed of a Hamilton syringe lowered into the orifice. As AD mice models express human APP transgenes, we decided to test human plasma FH (plFH, Sigma-Aldrich, Saint-Quentin-Fallavier, France) as it contains all post-translational modifications. Complement factor H was derived from human plasma (1 mg/mL in PBS, PH 7.2, >90% (SDS-Page) and was previously tested for infectious agents (Sigma-Aldrich, France). A solution containing plFH (5 μL of 50 μg diluted in a phosphate-buffered saline (PBS), Sigma-Aldrich, France) or PBS (5 μL) was infused over 3 min. The injector was then reassembled and the skull skin incision was sutured. This injected dose corresponded to approximately 5 to 10 times the quantity of FH detected in mouse brain volumes. A higher dose could create an immunodeficient microenvironment. Prior to the efficacy evaluation of FH, the diffusion of histidine-labeled FH (5 μL of 50 μg, Sigma-Aldrich, France) injected into the CSF was studied in the brain at 24 h, 1 month and 3 months post-injection. Stereotaxic coordinates for the injection were (relative to the bregma) 1 mm posterior, +/−0.5 mm lateral and 2 mm ventral. Each experimental group included a group with plFH injection (*n* = 10 to 15 animals) and a control group with PBS injection (*n* = 10 to 15 animals). The cognitive functions of these animals were assessed through water maze experiments (*n* = 10 to 15 mice), and immunostaining/Western blotting investigations were performed (*n* = 3 to 10 mice).

### 4.3. Optical Clearing:

For organ pretreatment and MAC immunolabeling, the iDISCO+ optical clearing protocol was adapted from existing protocols [60,61]. Briefly, each mouse received a retroorbital intravenous injection of 100 µL of lectin coupled to a fluorophore (Lycopersicon esculentum Lectin DyLight649^®^ 1 mg/mL, Vector Labs, Eurobio Scientific, Les Ulis, France) to stain the systemic capillary network. Ten minutes after the injection, a 100 µL intraperitoneal injection of isosorbide dinitrate was administered to dilate the vessels. The mouse was then euthanized by an intraperitoneal injection of 300 µL of sodium pentobarbital diluted in a physiological saline solution. After death, a sternotomy was carried out to catheterize the heart’s left ventricle. A perfusion of physiological solution at 80 mm Hg pressure for 3 min was conducted to remove the blood from the vasculature. A second perfusion with 4% formalin was performed to fix the tissues. The brain was delicately removed and placed in 4% paraformalin (PFA4%) overnight at 4 °C.

The brain was dehydrated by successive immersions in 20%, 40%, 60%, 80% and 100% methanol solutions, each for 1 h at room temperature (RT), followed by an overnight immersion at RT in a 2/3 dichloromethane and 1/3 methanol solution. Bleaching was performed in 5% H_2_O_2_ and 95% methanol overnight at 4 °C. The brain was rehydrated by successive immersions in 80%, 60%, 40% and 20% methanol solutions and PBS for 1 h each at RT.

For immunolabeling, permeabilization was performed by incubating the brain in a permeabilization solution (PBS Triton 0.5%) for 2 days at RT. Nonspecific antigen sites were blocked by incubation in a blocking solution (PBS/Triton 0.5%/normal goat serum 10%) for 3 days at RT. Immunolabeling steps were performed by incubating the brain in anti-C5b-9 or MAC (1:200 dilution in PBS/Triton 0.5%, Santa Cruz, Nanterre, France) primary antibody solution for 7 days at 4 °C under agitation, followed by incubation in TRITC (1:250 dilution in PBS/Triton 0.5%, Invitrogen, Illkirch, France), the secondary antibody solution, for 5 days at 4 °C under agitation. The brain was treated by successive immersions in 20%, 40%, 60% and 80% methanol solutions for 1 h each at RT, then left overnight in pure methanol at RT. Permeabilization and lipid removal were conducted in a 2/3 dichloromethane and 1/3 methanol solution for 3 h at RT. The remaining methanol was removed by two 15 min baths in pure dichloromethane solutions at RT. Last, refractive index matching was performed by immersing the sample in a dibenzyl ether solution for 2 h at RT and keeping it at 4 °C until imaging.

For image acquisition (light sheet microscopy), each brain was mounted in an ethyl cinnamate solution adapted to the light sheet microscope. For ultramicroscopy, the system from LaVision BioTec (Bielefeld, Germany), equipped with 545 and 640 nm laser lines, an sCMOS Andor camera and a 0.5 NA 2× objective with a deeping lens with 6.3 zoom, was used. The tissue was laterally illuminated by three horizontal sheets with an exposure time of 200 ms. Emitted fluorescences were collected at 630 nm and 690 nm, respectively. Upon completion of the acquisition, the stack was automatically reconstructed in 16-bit format. Capillary network imaging was made by sampling of 1080 × 1280 × 300 µm parallelipipedic sections in the hippocampus zone of the brain. The system’s spatial resolution was 1 µm (x, y) and 4 µm (z). The step size used was 2 µm. Voxel dimensions were 0.5 µm (x, y) and 2 µm (z).

### 4.4. Western Blot Analysis

Brains were lysed in ice-cold lysis buffer (50 mM Tris-HCL, pH 7.5, 100 mM NaCl, 0.1% NP-40, 1% deoxycholate, 50 mM ß-glycerophosphate, 0.2 mM sodium orthovanadate, 50 mM sodium fluoride, 1 µg/mL leupeptin, 5 µM pepstatin, 20 klU/mL aprotinin, 1 mM phenylmethylsulfonyl fluoride) and centrifuged for 10 min at 10,000× *g* at 4 °C. Protein concentrations were determined using a BCA kit (Thermo Fisher Scientific, Illkirch, France). Brain lysates (*n* = 10) were mixed with 5× Laemmli buffer and heated for 5 min at 95 °C. Subsequently, they were resolved by SDS-PAGE (10% polyacrylamide gels), electroblotted onto a polyvinylidene difluoride (PVDF) membrane (Immobilon, Millipore, France) and probed with antibodies against complement proteins C3/C3 fragments, FH and actin (refer to Table 2 for details). After incubation with the secondary antibody (donkey anti-rat or donkey anti-rabbit or donkey anti-goat) conjugated to Alexa Fluor 700 or Alexa Fluor 800 (diluted 1:5000, Invitrogen, France), antibody binding was detected using the Odyssey Infrared Imaging System (LI-COR Biosciences, Bad Homburg, Germany). Protein loading quantity was controlled using mouse monoclonal anti-ß actin antibodies (Cell Signaling, Saint-Cyr-L’École, France) (see Table 2 for details). The levels of FH production were semiquantified by comparing the intensity of the bands of FH obtained to those of actin.

### 4.5. Immunohistochemistry on Mice Brain

To assess the expression levels of complement proteins (C3, C3a, C5b-9 (MAC) and FH) in the hippocampus of C57Bl/6J and APP/PS1 mice, immunohistochemistry experiments were performed at 6, 7, 9, 10, 12 and 15 months. Brains were promptly removed just after death, fixed in PFA4%-PBS1X solution overnight at 4 °C and cryoprotected in successive sucrose solutions (10, 20 and 30%) diluted in PBS1X before being stored at −80 °C. Transverse brain sections (14 µm thickness) were cut using a Microm HM550 cryostat (Microm, Walldorf, Germany) at −20 °C. The sections were mounted onto Polysine^®^ glass slides (Polysine^®^ Adhesion slides, Thermo Scientific, France) and then stored at −80 °C until further processing. For staining, nonspecific binding sites were blocked (10% normal horse serum, 0.5% Triton X-100, in PBS) for 1 h at RT. The sections were incubated with primary antibodies overnight (Table 2). Following PBS1X washing (3 times for 10 min at RT), sections were incubated with the appropriate secondary antibodies (donkey anti-rabbit, donkey anti-mouse, donkey anti-rat or donkey anti-goat) conjugated to Alexa Fluor 488, Alexa Fluor 594 or Alexa Fluor 643 (diluted 1:500, Invitrogen, France), as needed, for 1 h at RT. After washing, sections were mounted with a fluorescent aqueous mounting medium containing DAPI (Dako, Les Ulis, France). For semiquantifications, images were acquired with a confocal microscope (LSM 700 Carl Zeiss, Fougères, France) using a 20× objective at a resolution of 1024 × 1024 pixels. All immunostaining experiments were repeated at least three to seven times with different animals. Nine nonoverlapping fields of linear hippocampus length were photographed at 20× magnification (Title 3 × 3). All images were obtained using the same parameters (pinhole, detector gain, amplifier offset, amplifier gain and laser intensity). The mean intensity (mean gray value, within the range from 0 to 255) of green, red, pink or white fluorescence in title 3 × 3 images was semiquantitatively analyzed with the ImageJ image-analyzing program (NIH, Bethesda, MD, USA). Data were expressed as relative immunoreactivity, representing the intensity of the immunoreaction relative to background staining and thereafter the mean of the three to seven animals was calculated. Blood–brain barrier permeability analysis was conducted through immunostaining experiments with an anti-albumin antibody, and images were acquired with a confocal microscope (LSM 700 Carl Zeiss, France) using a 63X/1.3 oil immersion objective at the same resolution described above. *n* = 5 to 7 mice were used for these experiments. Concerning the kinetics analysis of Aβ production in APP/PS1 mice, because WT mice do not produce human Aβ and the antibody used (6E10) recognizes human Aβ, we considered the Aβ signal observed in WT mice tissue as zero (antibody background), and then we subtracted this signal level from the APP/PS1 signal level.

### 4.6. Morris Water Maze Experiments

The Morris water maze (MWM) test is a widely used measure of visuospatial memory and learning. It has demonstrated strong validity in investigating the cognitive effects of various brain disorders and drugs used to treat cognitive deficits. The MWM test consists of a white circular pool (180 cm × 80 cm high) filled with water that is colored opaque with powdered non-fat milk to a depth of 35 cm at a temperature of 26 +/− 1 °C. The pool is imaginarily divided into four equal compartments named A to D. A platform for escape is randomly placed in one of the four quadrants. In the first step, named visualization tests, the animals see the platform without external/extra-maze cues. Here, the animals learn to find the visual platform more quickly. In the second step, named the training, the platform is submerged 1 cm below the surface of the water and four visual cues with different shapes are randomly designated on north, south, east and west, respectively. The mice must swim to the hidden escape platform. Subsequently, the mice are trained for 3 days, starting at four different regions (A, B, C and D) of the pool each day. For each trial and mouse, the latency time to find the submerged platform is recorded. If mice reach the platform in 60 s (s), they stay on it for 10 s; if not, they are guided to the platform passively by the experimenter and are allowed to rest on the platform for 30 s. In the protest session, mice are placed in the B region (opposite the ghost platform) and are allowed to navigate for 90 s without the platform. Here, only visual cues are provided. The experiments are monitored by a video camera placed above the middle of the pool. Latency time and cumulative duration are then recorded and analyzed, with *n* = 10 to 15 mice per treatment and strain mice.

### 4.7. Statistics

Immunostaining and Western blot experiments: The results were expressed as the immunostaining intensity ± the standard error of the mean (SEM). The spatial frequency threshold across ages (6,7,9,10, 12 and 15 months) and genotypes (APP/PS1 vs. C57BL/6J or APP/PS1) was analyzed using the Mann–Whitney U test (GraphPad Software version 10.0.). *p*-values of 0.05 or less were considered significant. Thus, in all figures, statistical significance was expressed as °^,^* *p* < 0.05, °°^,^** *p* < 0.01 and °°°^,^*** *p* < 0.001. *n* = 3 to 7 mice for immunostaining experiments and *n* = 10 for Western blot experiments.

For MWM experiments: The results were expressed as the time to reach or search the visible or invisible platform. All statistical analyses were performed with the GraphPad Prism program. Data were subjected to an analysis of variance (2-way ANOVA) followed by a post hoc test. Values were expressed as mean ± SEM. A *p*-value < 0.05 was acknowledged for a minimal significance level. °^,^* *p* < 0.05, °°^,^** *p* < 0.01 and °°°^,^*** *p* < 0.001. *n* = 10 to 15 mice per treatment (plFH or PBS) and strain mice (APP/PS1, WT, APP/PS1-FHko).

## 5. Conclusions

Unfortunately, real treatments to halt the pathophysiological processes or progression of AD are still unavailable. Our study introduces the possibility of considering FH as a promising therapeutic target to treat and slow down its progression (Figure 8). In the current landscape of therapeutic research for AD, regulating complement activation could unveil a new drug molecule applicable to both the early and late stages of this pathology. This stands in contrast to ongoing research (donanemab and lecanemab) [7,8], which predominantly target Aβ deposits and are generally effective only for patients with mild cognitive impairment or in the early stages of the disease. Moreover, regulating the inflammatory reaction in AD by reducing complement activation not only diminishes the formation of Aβ plaques but also mitigates damaging effects on synaptic transmission and cognitive functions, as evidenced by our results. Thus, complement therapy has the potential to concurrently address multiple processes contributing to the worsening of AD, in contrast to treatment strategies focusing solely on anti-Aβ targeting. Importantly, FH offers a significant advantage as it is naturally produced by the organism, making it less likely to induce toxicity compared to the current use of antibodies specifically directed against Aβ, which may lead to brain edema in patients.

## Figures and Tables

**Table 1:** Summary of the effects of plFH injection on cognitive functions (cumulative duration and latency to first) in APP/PS1 mice. The percentage increase or decrease in cognitive functions following plFH or PBS brain injection in APP/PS1mice was obtained after comparison with the cognitive function values of WT mice during both early (6 months + 1 or 3 months) and late stages (6 months +1, +3 or +6 months). The study involved 10 to 15 animals for each experimental group (APP/PS1, plFH-injected APP/PS1 mice (+FH), PBS-injected APP/PS1 mice (+PBS) and WT mice).

Stage (Months M)	WT	APP/PS1	+PBS	+FH
6 M + 1 M	2.61	0.77	1.1	3.03
		↓70%	↓60%	=
6 M + 3 M	4.27	1.623	1.615	1.265
		↓62%	↓63%	↓70%
9 M + 1 M	4.267	1.29	0.88	2.721
		↓70%	↓75%	↓36%
9 M + 3 M	2.55	0.61	1.011	2.851
		↓72%	↓61%	=
9 M + 6 M	2.211	0.62	0.64	1.573
		↓73%	↓72%	↓30%
**Cumulative duration (s)**				
6M + 1M	16.47	58.5	51.37	22.81
		↑3.5%	↑3.1%	↑1.3%
6M + 3M	16.59	46.29	39.71	34.94
		↑2.8%	↑2.4%	↑2.1%
9M + 1M	18.68	71.33	88.46	29.45
		↑3.8%	↑4.7%	↑1.6%
9M + 3M	21.3	69.19	61.5	18.37
		↑3.2%	↑2.8%	= (0.8)
9M + 6M	16.5	65.61	50.07	31.35
		↑3.7%	↑3%	↑2%
**Latency to first (s)**				

**Table 2:** List of antibodies used for Western blot, immunostaining and injection experiments.

Western Blot	Host	Dilution	Manufacturer
C3/C3b fragments	Rat	1/1000	Hycult
FH	Goat	1/1500	Quidel
Actin-β	Rabbit	1/1500	Santa Cruz
**Immunostaining**	**Host**	**Dilution**	**Manufacturer**
C3	Rabbit	1/500	Abcam
C3a	Rat	1/400	DB Pharmingen
C5b-9-FITC	-	1/500	Santa Cruz
FH	Goat	1/600	Quidel
Albumin	Sheep	1/600	Abcam
Cd31	Mouse	1/700	Chemicon
VGLUT1	Mouse	1/500	Santa Cruz
Psd95-FITC	-	1/400	Santa Cruz
Synapsin	Mouse	1/400	Santa Cruz
NeuN	Rabbit	1/500	Millipore
IL6	Goat	1/500	R&D Systems
IL1β	Goat	1/500	R&D Systems
TNF-α	Rat	1/500	Biolegend
MMR2	Mouse	1/600	Millipore
Aβ(6E10)	Mouse	1/400	Biolegend
**Injection**	**-**	**Quantity**	**Manufacturer**
Human plFH	-	50 µg/5 µL	Sigma-Aldrich
FH-His Tag	-	50 µg/5 µL	Sigma-Aldrich

## Data Availability

The data presented in this study are available on request from the corresponding author (accurately indicate status).

## Supplementary Materials

The supporting information can be downloaded at: https://www.mdpi.com/article/10.3390/ijms25042272/s1.

## References

[1] PubMed Alzheimer’s Disease. https://pubmed.ncbi.nlm.nih.gov/20107219/.

[2] Scheff S.W., Price D.A., Schmitt F.A., Mufson E.J. (2006). Hippocampal synaptic loss in early Alzheimer’s disease and mild cognitive impairment. Neurobiol. Aging.

[3] Reddy P.H., Mani G., Park B.S., Jacques J., Murdoch G., Whetsell W., Kaye J., Manczak M. (2005). Differential loss of synaptic proteins in Alzheimer’s disease: Implications for synaptic dysfunction. J. Alzheimer’s Dis..

[4] Gong Y., Lippa C.F., Zhu J., Lin Q., Rosso A.L. (2009). Disruption of glutamate receptors at Shank-postsynaptic platform in Alzheimer’s disease. Brain Res..

[5] Briggs R., Kennelly S.P., O’Neill D. (2016). Drug treatments in Alzheimer’s disease. Clin. Med..

[6] Schenk D., Basi G.S., Pangalos M.N. (2012). Treatment Strategies Targeting Amyloid β-Protein. Cold Spring Harb. Perspect. Med..

[7] van Dyck C.H., Swanson C.J., Aisen P., Bateman R.J., Chen C., Gee M., Kanekiyo M., Li D., Reyderman L., Cohen S. (2023). Lecanemab in Early Alzheimer’s Disease. N. Engl. J. Med..

[8] Gueorguieva I., Willis B.A., Chua L., Chow K., Ernest C.S., Shcherbinin S., Ardayfio P., Mullins G.R., Sims J.R. (2023). Donanemab Population Pharmacokinetics, Amyloid Plaque Reduction, and Safety in Participants with Alzheimer’s Disease. Clin. Pharmacol. Ther..

[9] Selkoe D.J., Hardy J. (2016). The amyloid hypothesis of Alzheimer’s disease at 25 years. EMBO Mol. Med..

[10] Sánchez-Rodríguez I., Djebari S., Temprano-Carazo S., Vega-Avelaira D., Jiménez-Herrera R., Iborra-Lázaro G., Yajeya J., Jiménez-Díaz L., Navarro-López J.D. (2019). Hippocampal long-term synaptic depression and memory deficits induced in early amyloidopathy are prevented by enhancing G-protein-gated inwardly rectifying potassium channel activity. J. Neurochem..

[11] Kim H.Y., Lee D.K., Chung B.-R., Kim H.V., Kim Y. (2016). Intracerebroventricular Injection of Amyloid-β Peptides in Normal Mice to Acutely Induce Alzheimer-like Cognitive Deficits. J. Vis. Exp..

[12] Beason-Held L.L., Goh J.O., An Y., Kraut M.A., O’Brien R.J., Ferrucci L., Resnick S.M. (2013). Changes in Brain Function Occur Years before the Onset of Cognitive Impairment. J. Neurosci..

[13] Aisen P.S., Cummings J., Jack C.R., Morris J.C., Sperling R., Frölich L., Jones R.W., Dowsett S.A., Matthews B.R., Raskin J. (2017). On the path to 2025: Understanding the Alzheimer’s disease continuum. Alzheimer’s Res. Ther..

[14] Eikelenboom P., Stam F.C. (1982). Immunoglobulins and complement factors in senile plaques. An immunoperoxidase study. Acta Neuropathol..

[15] McGeer P.L., McGeer E.G. (1995). The inflammatory response system of brain: Implications for therapy of Alzheimer and other neurodegenerative diseases. Brain Res. Rev..

[16] McGeer P.L., Rogers J., McGeer E.G. (2016). Inflammation, Antiinflammatory Agents, and Alzheimer’s Disease: The Last 22 Years. J. Alzheimer’s Dis..

[17] Kolev M., Ruseva M., Harris C., Morgan B., Donev R. (2009). Implication of Complement System and its Regulators in Alzheimers Disease. Curr. Neuropharmacol..

[18] Yasojima K., Schwab C., McGeer E.G., McGeer P.L. (1999). Up-Regulated Production and Activation of the Complement System in Alzheimer’s Disease Brain. Am. J. Pathol..

[19] Bellenguez C., Küçükali F., Jansen I., Andrade V., Moreno-Grau S., Amin N., Naj A.C., Grenier-Boley B., Campos-Martin R., Holmans P.A. (2020). New Insights on the Genetic Etiology of Alzheimer’s and Related Dementia. Nat. Genet..

[20] Torvell M., Carpanini S.M., Daskoulidou N., Byrne R.A.J., Sims R., Morgan B.P. (2021). Genetic Insights into the Impact of Complement in Alzheimer’s Disease. Genes.

[21] Zhang D.-F., Li J., Wu H., Cui Y., Bi R., Zhou H.-J., Wang H.-Z., Zhang C., Wang D., Alzheimer’s Disease Neuroimaging Initiative (ADNI) (2015). CFH Variants Affect Structural and Functional Brain Changes and Genetic Risk of Alzheimer’s Disease. Neuropsychopharmacology.

[22] Zetterberg M., Landgren S., Andersson M.E., Palmér M.S., Gustafson D.R., Skoog I., Minthon L., Thelle D.S., Wallin A., Bogdanovic N. (2008). Association of complement factor H Y402H gene polymorphism with Alzheimer’s disease. Am. J. Med. Genet. Part B Neuropsychiatr. Genet..

[23] Le Fur I., Laumet G., Richard F., Fievet N., Berr C., Rouaud O., Delcourt C., Amouyel P., Lambert J.-C. (2010). Association study of the CFH Y402H polymorphism with Alzheimer’s disease. Neurobiol. Aging.

[24] Borras C., Canonica J., Jorieux S., Abache T., El Sanharawi M., Klein C., Delaunay K., Jonet L., Salvodelli M., Naud M.-C. (2019). CFH exerts anti-oxidant effects on retinal pigment epithelial cells independently from protecting against membrane attack complex. Sci. Rep..

[25] Borras C., Delaunay K., Slaoui Y., Abache T., Jorieux S., Naud M.-C., El Sanharawi M., Gelize E., Lassiaz P., An N. (2020). Mechanisms of FH Protection Against Neovascular AMD. Front. Immunol..

[26] Wu T., Dejanovic B., Gandham V.D., Gogineni A., Edmonds R., Schauer S., Srinivasan K., Huntley M.A., Wang Y., Wang T.-M. (2019). Complement C3 Is Activated in Human AD Brain and Is Required for Neurodegeneration in Mouse Models of Amyloidosis and Tauopathy. Cell Rep..

[27] Shi Q., Chowdhury S., Ma R., Le K.X., Hong S., Caldarone B.J., Stevens B., Lemere C.A. (2017). Complement C3 deficiency protects against neurodegeneration in aged plaque-rich APP/PS1 mice. Sci. Transl. Med..

[28] An X.-Q., Xi W., Gu C.-Y., Huang X. (2018). Complement protein C5a enhances the β-amyloid-induced neuro-inflammatory response in microglia in Alzheimer’s disease. Med. Sci..

[29] Mukherjee P., Pasinetti G.M. (2000). The role of complement anaphylatoxin C5a in neurodegeneration: Implications in Alzheimer’s disease. J. Neuroimmunol..

[30] Panayiotou E., Fella E., Andreou S., Papacharalambous R., Gerasimou P., Costeas P., Angeli S., Kousiappa I., Papacostas S., Kyriakides T. (2019). C5aR agonist enhances phagocytosis of fibrillar and non-fibrillar Aβ amyloid and preserves memory in a mouse model of familial Alzheimer’s disease. PLoS ONE.

[31] Ordoñez-Gutierrez L., Fernandez-Perez I., Herrera J.L., Anton M., Benito-Cuesta I., Wandosell F. (2016). AβPP/PS1 Transgenic Mice Show Sex Differences in the Cerebellum Associated with Aging. J. Alzheimer’s Dis..

[32] Wang J., Tanila H., Puoliväli J., Kadish I., van Groen T. (2003). Gender differences in the amount and deposition of amyloidβ in APPswe and PS1 double transgenic mice. Neurobiol. Dis..

[33] Vinters H.V. (1987). Cerebral amyloid angiopathy. A critical review. Stroke.

[34] Charidimou A., Gang Q., Werring D.J. (2011). Sporadic cerebral amyloid angiopathy revisited: Recent insights into pathophysiology and clinical spectrum. J. Neurol. Neurosurg. Psychiatry.

[35] Krstic D., Knuesel I. (2012). Deciphering the mechanism underlying late-onset Alzheimer disease. Nat. Rev. Neurol..

[36] Cavanagh C., Colby-Milley J., Bouvier D., Farso M., Chabot J.-G., Quirion R., Krantic S. (2013). βCTF-Correlated Burst of Hippocampal TNFα Occurs at a Very Early, Pre-Plaque Stage in the TgCRND8 Mouse Model of Alzheimer’s Disease. J. Alzheimer’s Dis..

[37] Cavanagh C., Tse Y.C., Nguyen H.-B., Krantic S., Breitner J.C., Quirion R., Wong T.P. (2016). Inhibiting tumor necrosis factor-α before amyloidosis prevents synaptic deficits in an Alzheimer’s disease model. Neurobiol. Aging.

[38] Dinet V., Arouche-Delaperche L., Dégardin J., Naud M.-C., Picaud S., Krantic S. (2022). Concomitant Retinal Alterations in Neuronal Activity and TNFα Pathway Are Detectable during the Pre-Symptomatic Stage in a Mouse Model of Alzheimer’s Disease. Cells.

[39] Doméné A., Cavanagh C., Page G., Bodard S., Klein C., Delarasse C., Chalon S., Krantic S. (2016). Expression of Phenotypic Astrocyte Marker Is Increased in a Transgenic Mouse Model of Alzheimer’s Disease versus Age-Matched Controls: A Presymptomatic Stage Study. Int. J. Alzheimer’s Dis..

[40] Decourt B., Lahiri D.K., Sabbagh M.N. (2016). Targeting Tumor Necrosis Factor Alpha for Alzheimer’s Disease. Curr. Alzheimer Res..

[41] Liu J., Tan Y., Zhang J., Zou L., Deng G., Xu X., Wang F., Ma Z., Zhang J., Zhao T. (2015). C5aR, TNF-α, and FGL2 contribute to coagulation and complement activation in virus-induced fulminant hepatitis. J. Hepatol..

[42] Forloni G., Demicheli F., Giorgi S., Bendotti C., Angeretti N. (1992). Expression of amyloid precursor protein mRNAs in endothelial, neuronal and glial cells: Modulation by interleukin-1. Mol. Brain Res..

[43] Li Y., Liu L., Barger S.W., Griffin W.S.T. (2003). Interleukin-1 Mediates Pathological Effects of Microglia on Tau Phosphorylation and on Synaptophysin Synthesis in Cortical Neurons through a p38-MAPK Pathway. J. Neurosci..

[44] Kitazawa M., Cheng D., Tsukamoto M.R., Koike M.A., Wes P.D., Vasilevko V., Cribbs D.H., LaFerla F.M. (2011). Blocking IL-1 Signaling Rescues Cognition, Attenuates Tau Pathology, and Restores Neuronal β-Catenin Pathway Function in an Alzheimer’s Disease Model. J. Immunol..

[45] Huell M., Strauss S., Volk B., Berger M., Bauer J. (1995). Interleukin-6 is present in early stages of plaque formation and is restricted to the brains of Alzheimer’s disease patients. Acta Neuropathol..

[46] Walsh K.P., Minamide L.S., Kane S.J., Shaw A.E., Brown D.R., Pulford B., Zabel M.D., Lambeth J.D., Kuhn T.B., Bamburg J.R. (2014). Amyloid-β and Proinflammatory Cytokines Utilize a Prion Protein-Dependent Pathway to Activate NADPH Oxidase and Induce Cofilin-Actin Rods in Hippocampal Neurons. PLoS ONE.

[47] Heyser C.J., Masliah E., Samimi A., Campbell I.L., Gold L.H. (1997). Progressive decline in avoidance learning paralleled by inflammatory neurodegeneration in transgenic mice expressing interleukin 6 in the brain. Proc. Natl. Acad. Sci. USA.

[48] e Silva N.M.L., Gonçalves R.A., Pascoal T.A., Lima-Filho R.A.S., Resende E.d.P.F., Vieira E.L.M., Teixeira A.L., de Souza L.C., Peny J.A., Fortuna J.T.S. (2021). Pro-inflammatory interleukin-6 signaling links cognitive impairments and peripheral metabolic alterations in Alzheimer’s disease. Transl. Psychiatry.

[49] Swardfager W., Lanctôt K., Rothenburg L., Wong A., Cappell J., Herrmann N. (2010). A Meta-Analysis of Cytokines in Alzheimer’s Disease. Biol. Psychiatry.

[50] Terry R.D., Masliah E., Salmon D.P., Butters N., DeTeresa R., Hill R., Hansen L.A., Katzman R. (1991). Physical basis of cognitive alterations in alzheimer’s disease: Synapse loss is the major correlate of cognitive impairment. Ann. Neurol..

[51] Fayed N., Modrego P.J., Rojas-Salinas G., Aguilar K. (2011). Brain Glutamate Levels Are Decreased in Alzheimer’s Disease. Am. J. Alzheimer’s Dis. Other Dementiasr.

[52] Johnson J., Sherry D.M., Liu X., Fremeau R.T., Seal R.P., Edwards R.H., Copenhagen D.R. (2004). Vesicular glutamate transporter 3 expression identifies glutamatergic amacrine cells in the rodent retina. J. Comp. Neurol..

[53] Kashani A., Lepicard E., Poirel O., Videau C., David J.P., Fallet-Bianco C., Simon A., Delacourte A., Giros B., Epelbaum J. (2008). Loss of VGLUT1 and VGLUT2 in the prefrontal cortex is correlated with cognitive decline in Alzheimer disease. Neurobiol. Aging.

[54] Rodriguez-Perdigon M., Tordera R.M., Gil-Bea F.J., Gerenu G., Ramirez M.J., Solas M. (2016). Down-regulation of glutamatergic terminals (VGLUT1) driven by Aβ in Alzheimer’s disease. Hippocampus.

[55] Rodriguez-Perdigon M., Solas M., Ramirez M.J. (2016). JNK: A Putative Link between Insulin Signaling and VGLUT1 in Alzheimer’s Disease. J. Alzheimer’s Dis..

[56] Proctor D.T., Coulson E.J., Dodd P.R. (2010). Reduction in Post-Synaptic Scaffolding PSD-95 and SAP-102 Protein Levels in the Alzheimer Inferior Temporal Cortex is Correlated with Disease Pathology. J. Alzheimer’s Dis..

[57] Shi Q., Colodner K.J., Matousek S.B., Merry K., Hong S., Kenison J.E., Frost J.L., Le K.X., Li S., Dodart J.-C. (2015). ComplementC3-Deficient Mice Fail to Display Age-Related Hippocampal Decline. J. Neurosci..

[58] Laskowski J., Renner B., Pickering M.C., Serkova N.J., Smith-Jones P.M., Clambey E.T., Nemenoff R.A., Thurman J.M. (2020). Complement factor H–deficient mice develop spontaneous hepatic tumors. J. Clin. Investig..

[59] Podcasy J.L., Epperson C.N. (2016). Considering sex and gender in Alzheimer disease and other dementias. Dialog. Clin. Neurosci..

[60] Renier N., Adams E.L., Kirst C., Wu Z., Azevedo R., Kohl J., Autry A.E., Kadiri L., Venkataraju K.U., Zhou Y. (2016). Mapping of Brain Activity by Automated Volume Analysis of Immediate Early Genes. Cell.

[61] Nicolas N., Roux E. (2021). 3D Imaging and Quantitative Characterization of Mouse Capillary Coronary Network Architecture. Biology.

