# Supplementary material for: Factor H’s Control of Complement Activation Emerges as a Significant and Promising Therapeutic Target for Alzheimer’s Disease Treatment
Source: Int J Mol Sci. 2024 Feb 14;25(4):2272. doi: 10.3390/ijms25042272 (PMC10889136; doi:10.3390/ijms25042272)
Supplement: Supplementary file 1 [file ijms-25-02272-s001.zip › ijms-2855746-supplementary.pdf]

Supplemental data :

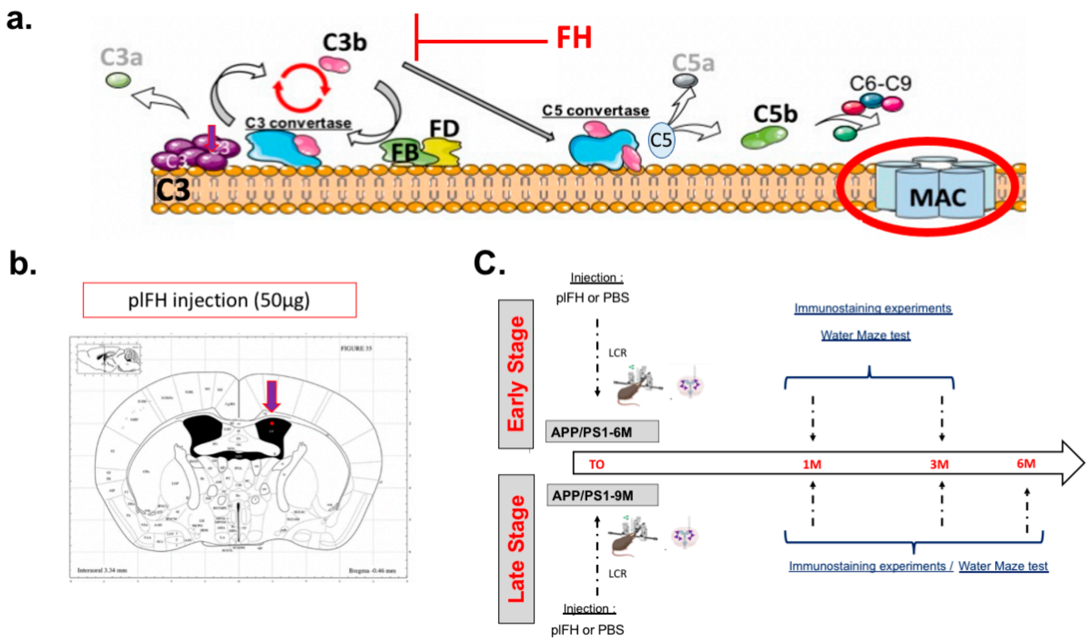

Supplemental Figure S1: (a.) Schematic representation of the alternative complement pathway **(b.)** Stereotaxic coordinates for the injection are (relative to the bregma): 1 mm posterior, +/-0.5 mm lateral and 2 mm ventral. Each experimental group included a group with plFH injection (n=10 to 15 animals) and a control group with PBS injection (n=10 to 15 animals) **(c.)** Protocol used for plFH injection effects analysis **(**n=10 to 15 animals per experimental groups injected with plFH or PBS).

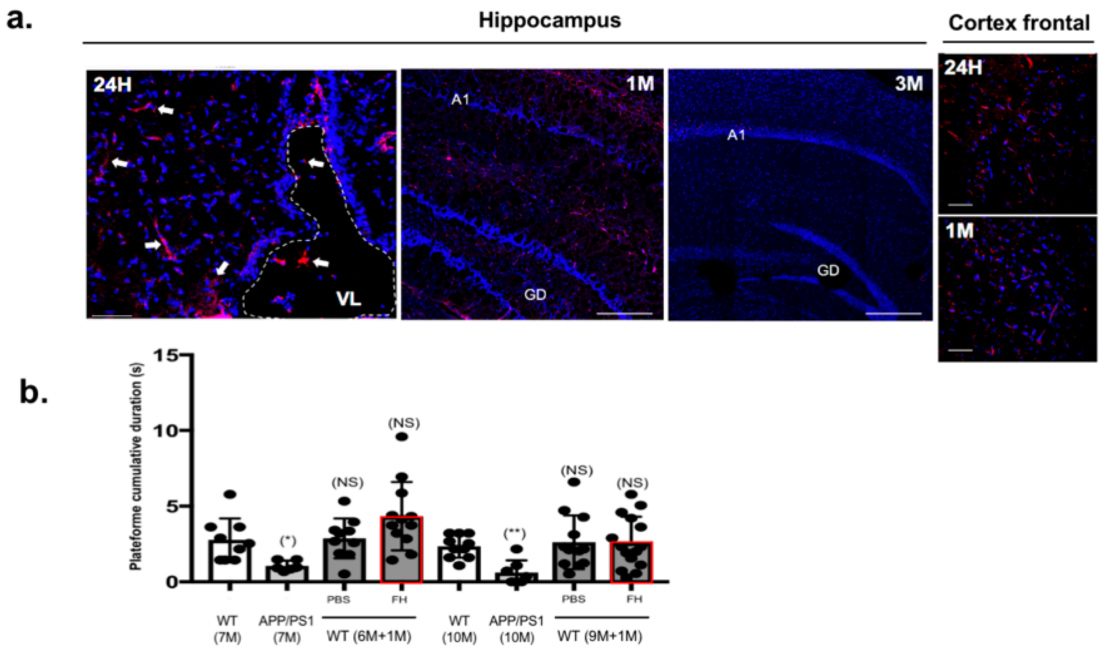

Supplemental Figure S2: (a.) Analysis by immunostaining experiments of FH-His-TAG injected diffusion (red) in the hippocampus and frontal cortex of WT mice at 24 hours, 1 month and 3 months post-injection **(**n=3 to 7 animals per experimental groups injected with plFH or PBS). GD=Gyrus Dentatus (dentate gyrus); VL=Lateral Ventricular. Scale bar=50µm. **(b.)** Cognitive functions were analyzed using Morris Water Maze experiments to assess plFH injection toxicity in WT mice (n=10 to 15 animals per experimental group, respectively injected with plFH or PBS). Each point represented a measure of one mouse's cognitive functions. Values were expressed as mean ± SEM. Data were subjected to an analysis of variance (2-way ANOVA) followed by the post hoc test. A *P* value < 0.05 was acknowledged for the minimal significance level. .* P<0.05; **P<0.01and NS=non-significant.

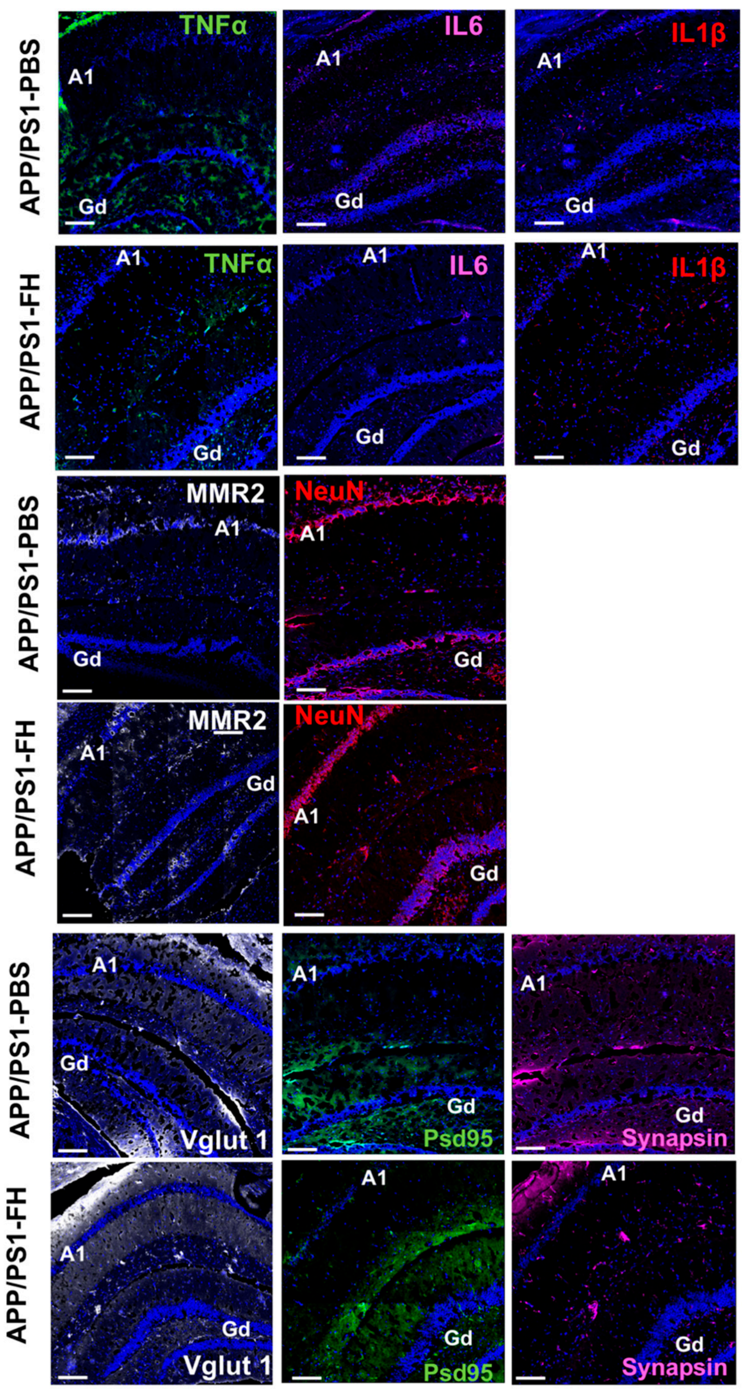

Supplemental Figure S3: Immunostaining images depicting inflammation markers (TNFα in green, IL6 in pink, IL1ß in red and MMR2 in white); **Neuronal cell survival marker** (NeuN in red), **and synaptic transmission analysis markers** (VGLUT1 in white, Psd95 in green and Synapsin in pink) in the hippocampus of plFH versus PBS-injected APP/PS1 mice (6M) one month post injection (presymptomatic stage). n=5 to 7 mice for each immunostaining experiment. Gd=Gyrus dentatus (dentate gyrus), Scale bar=50µm.

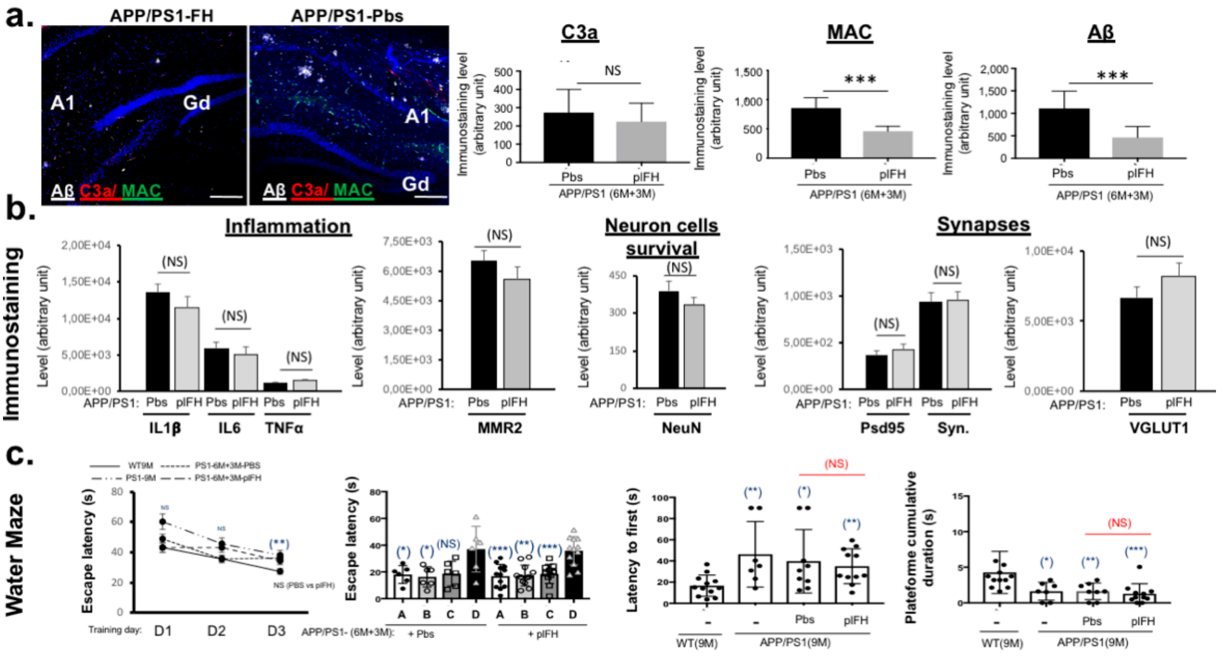

Supplemental Figure S4: Analysis of plFH effects 3 months after injection into the brains of APP/PS1 mice administered at 6 months (presymptomatic stage). Immunostaining analysis **(a.)** of complement activation markers (MAC, C3a and Aß deposits), **(b.)** of inflammation (IL1ß, IL6, TNFα, MMR2), Neuronal cell survival (NeuN) and synaptic transmission (VGLUT1, Psd95 and Synapsin (Syn.)) in the hippocampus of PBS or plFH-injected APP/PS1 mice (n=5 to 7 for each experimental group). Results were expressed as the immunostaining intensity ± the standard error of the mean (SEM). Spatial frequency threshold across injection (APP/PS1 injected with plFH or PBS) was analyzed by Mann-Whitney U test. P-values of 0.05 or less were considered significant. Gd=Gyrus dentatus (dentate gyrus), scale bar=50µm. **(c.)** Analysis of cognitive functions by Morris Water Maze experiments in plFH-injected APP/PS1 mice versus PBS-injected or versus APP/PS1 and WT mice. n=10 to 15 animals for each experimental group (APP/PS1 not injected mice, plFH-injected APP/PS1 mice, PBS-injected APP/PS1 mice and WT mice). Each point represented a measurement of one mouse. Data were subjected to an analysis of variance (2-way ANOVA) followed by the

post hoc test. Values were expressed as mean ± SEM. *P* value < 0.05 was acknowledged for minimal significance level. * P<0.05; **P<0.01;***P<0.005 and NS=non-significant.

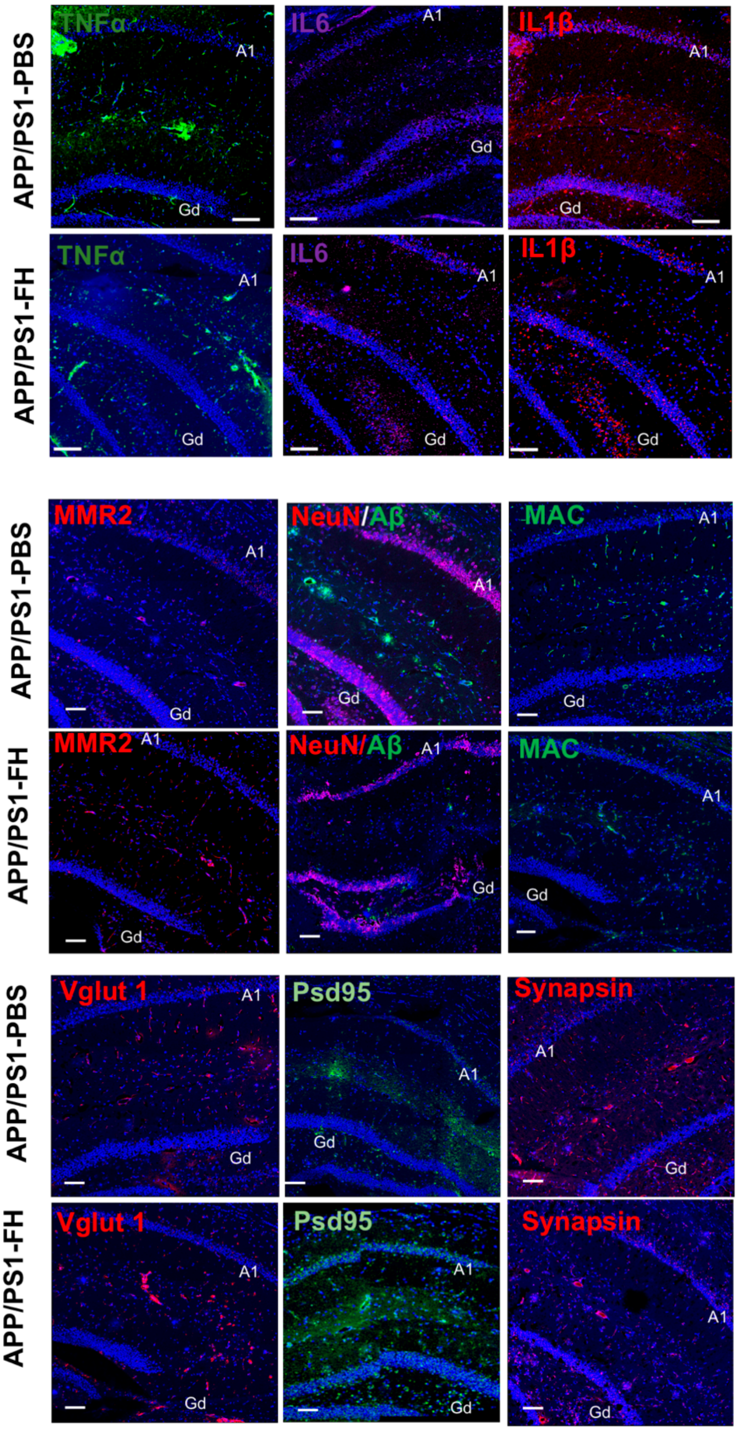

Supplemental Figure S5: Immunostaining images depicting Aß deposits (green); **Inflammation markers** (MAC in green, TNFα in green, IL6 in violet, IL1ß in red and MMR2

in red); **Neuronal cell survival marker** (NeuN in white) and **synaptic transmission markers** (VGLUT1 in red, Psd95 in green and Synapsin in red) in the hippocampus of plFH-injected versus PBS-injected APP/PS1 mice (9 months) observed at 3 months post injection (symptomatic stage). n=5 to 7 mice for each immunostaining experiment. Gd=Gyrus dentatus (dentate gyrus). Scale bar=50µm.

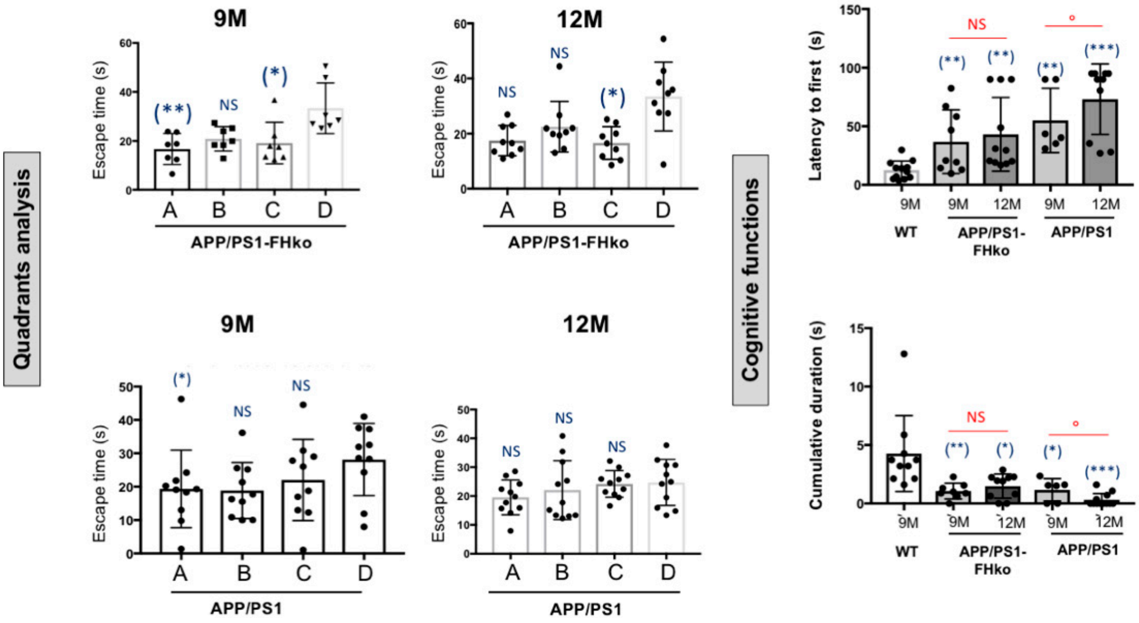

Supplemental Figure S6: Analysis of cognitive functions of APP/PS1-FH KO *versus* APP/PS1 mice at 9 and 12 months (Morris Water Maze experiments). n=10 to 15 animals for each experimental group (APP/PS1 mice, APP/PS1-FHko mice and WT mice). Each point represented a measurement of one mouse. Data were subjected to an analysis of variance (2-way ANOVA) followed by the post hoc test. Values were expressed as mean ± SEM. *P* value < 0.05 was acknowledged for the minimal significance level.* P<0.05; **P<0.01;***P<0.005 and NS=non-significant.

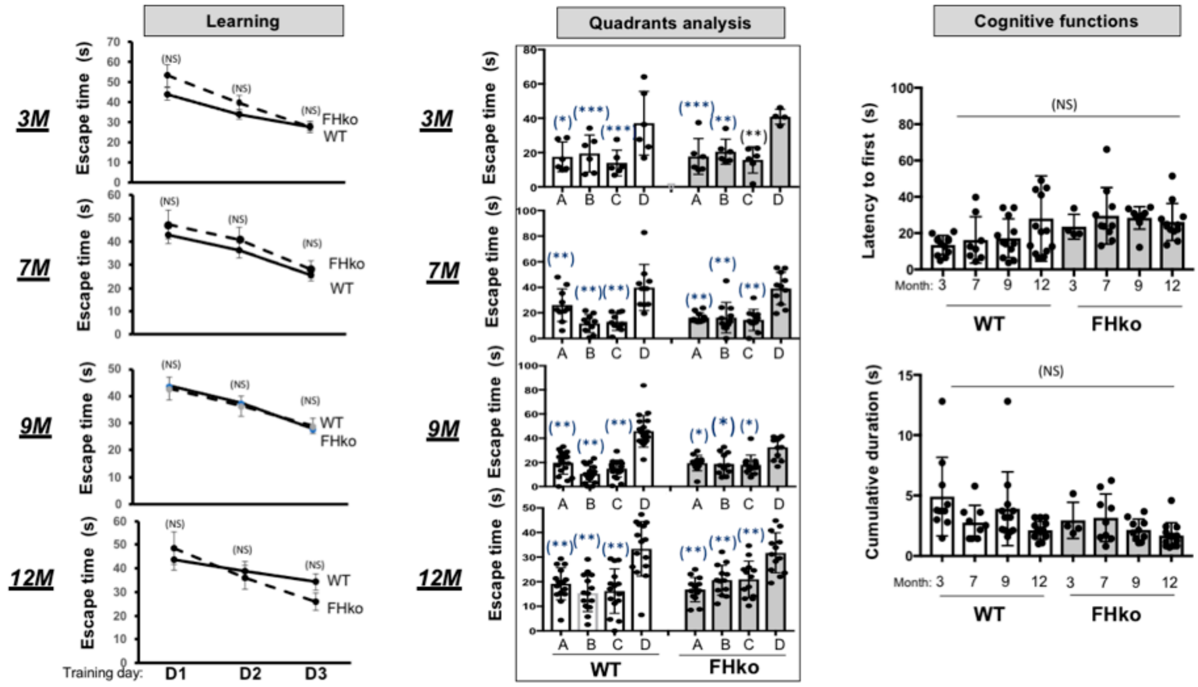

Supplemental Figure S7: Analysis of cognitive functions by Morris Water Maze experiments of FHko versus WT mice at 3, 7, 9 and 12 months. n=10 to 15 animals for each experimental group (FHko mice and WT mice (WT)). Each point represented a measurement of one mouse. Data were subjected to an analysis of variance (2-way ANOVA) followed by the post hoc test. Values were expressed as mean ± SEM. *P* value < 0.05 was acknowledged for the minimal significance level.* P<0.05; **P<0.01;***P<0.005 and NS=non-significant.
